# Inadvertent positioning of suprapubic catheter in urethra: a serious complication during change of suprapubic cystostomy in a spina bifida patient - a case report

**DOI:** 10.1186/1757-1626-2-9372

**Published:** 2009-12-22

**Authors:** Subramanian Vaidyanathan, Peter L Hughes, Bakul M Soni, Tun Oo, Gurpreet Singh

**Affiliations:** 1 Spinal Injuries Unit, District General Hospital, Town Lane, Southport PR8 6PN, UK; 2 Department of Radiology, District General Hospital, Southport PR8 6PN, UK; 3 Department of Urology, District General Hospital, Southport PR8 6PN, UK

## Abstract

**Introduction:**

Spinal cord injury patients are at risk for developing unusual complications such as autonomic dysreflexia while changing suprapubic cystostomy. We report a male patient with spina bifida in whom the Foley catheter was placed in the urethra during change of suprapubic cystostomy with serious consequences.

**Case presentation:**

A male patient, born in 1972 with spina bifida and paraplaegia, underwent suprapubic cystostomy in 2003 because of increasing problems with urethral catheter. The patient would come to spinal unit for change of suprapubic catheter every four to six weeks. Two days after a routine catheter change in November 2009, this patient woke up in the morning and noticed that the suprapubic catheter had come out. He went straight to Accident and Emergency. The suprapubic catheter was changed by a health professional and this patient was sent home. But the suprapubic catheter did not drain urine. This patient developed increasing degree of pain and swelling in suprapubic region. He did not pass any urine per urethra. He felt sick and came to spinal unit five hours later. About twenty ml of contrast was injected through suprapubic catheter and X-rays were taken. The suprapubic catheter was patent; the catheter was not blocked. The Foley catheter could be seen going around in a circular manner through the urinary bladder into the urethra. The contrast did not opacify urinary bladder; but proximal urethra was seen. The tip of Foley catheter was lying in proximal urethra. The balloon of Foley catheter had been inflated in urethra. When the balloon of Foley catheter was deflated, this patient developed massive bleeding per urethra. A sterile 22 French Foley catheter was inserted through suprapubic track. The catheter drained bloody urine. He was admitted to spinal unit and received intravenous fluids and meropenem. Haematuria subsided after 48 hours. The patient was discharged home a week later in a stable condition.

**Conclusion:**

This case shows that serious complications can occur during change of suprapubic catheter in patients with neuropathic bladder. After inserting a new catheter, health professionals should observe spinal cord injury patients for at least thirty minutes and ensure that (1) suprapubic catheter drains clear urine; (2) patients do not develop abdominal spasm or discomfort; (3) symptoms and signs of sepsis or autonomic dysreflexia are absent.

## Background

Unusual complications may be observed while changing a suprapubic catheter in patients with neuropathic bladder. For example, spinal cord injury patients with lesions above T-6 may develop autonomic dysreflexia [1]. Some patients may have widely open bladder neck because of previous bladder neck resection. In patients, who had long-term indwelling catheters, and in whom catheter care had been less than satisfactory, the bladder neck might have become patulous as a consequence of traction upon Foley balloon. In these patients, while changing a suprapubic catheter, the Foley catheter can be inserted inadvertently into urethra through widely open bladder neck. If the balloon is then inflated, the lumen of urethra is likely to be occluded by the Foley balloon. The tip of Foley catheter will be lying in urethra distal to the balloon and therefore, the catheter will not drain urine from urinary bladder. A health professional may inject a small amount of sterile 0.9% sodium chloride solution through the catheter and the catheter will appear to be patent, thus giving a false impression that the catheter is fine.

We describe a male patient with spina bifida in whom the suprapubic catheter was inserted inadvertently in to the urethra and the patient was sent home from accident and emergency. This patient developed increasing lower abdominal discomfort and the catheter did not drain urine. When the balloon of Foley catheter was deflated, there was massive bleeding per urethra and this patient developed haematuria as well, which was due to distension of urinary bladder.

## Case presentation

A British, Caucasian, male patient, born in 1972 with spina bifida and paraplaegia, had been managing neuropathic bladder with an indwelling urethral Foley catheter of size 20 French. He would insert the Foley catheter per urethra and also remove the catheter himself. Over the years, this patient developed increasing problems with urinary catheter. He was bypassing the catheter. The catheter had eroded penile urethra. The catheter would get blocked and he would then leak urine. This patient was fed up with the urethral catheter. Therefore, suprapubic cystostomy was performed in September 2003. A week later, the suprapubic catheter came out spontaneously, as the balloon had become deflated. The suprapubic track had closed. It was not possible to insert even a guide wire. Therefore, a catheter was passed per urethra. Suprapubic cystostomy was performed again in June 2004. The patient would come to spinal unit for change of suprapubic catheter every four to six weeks. The last catheter change was on 09 November 2009, which was uneventful.

This patient woke up around 0400 hours on 11 November 2009 and noticed that the suprapubic catheter had come out. He went straight to Accident and Emergency. The suprapubic catheter was changed by a health professional and this patient was sent home from Accident and Emergency. But the suprapubic catheter did not drain urine. This patient developed increasing degree of pain in suprapubic region. He noticed a swelling in suprapubic region. In the past, when suprapubic catheter was blocked, this patient would pass urine per urethra. But on this occasion, he did not pass urine per urethra. He was visibly uncomfortable; he felt sick. He reported to spinal unit at 0930 hours.

**Figure 1:**
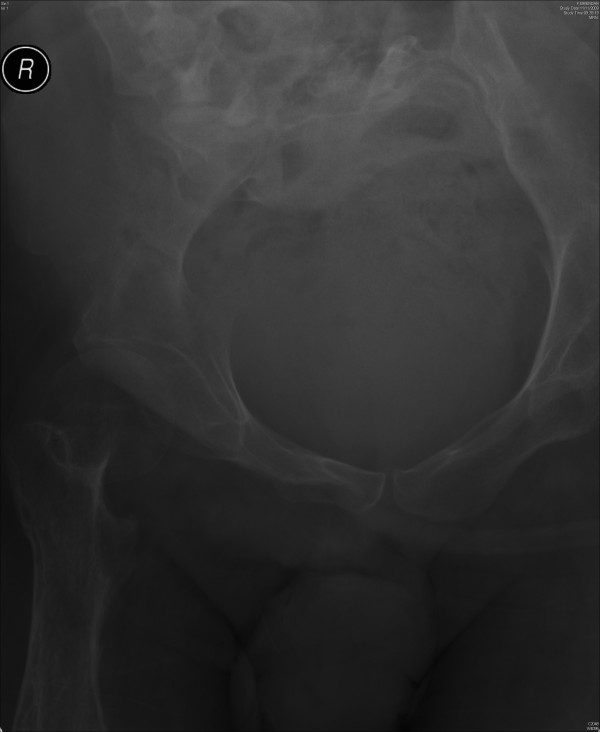
X-ray of pelvis, taken on 11 November 2009, showed soft tissue shadow of distended urinary bladder in pelvis.

**Figure 2:**
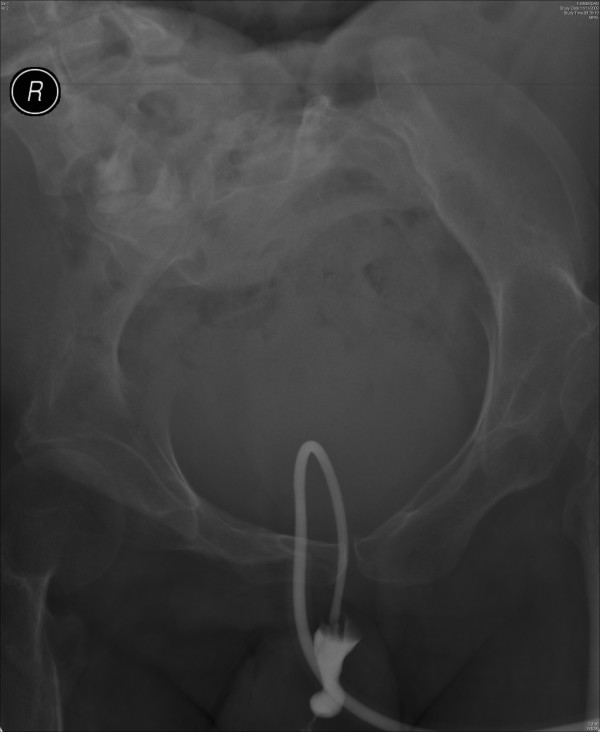
**Cystogram, performed on 11 November 2009, showed the suprapubic catheter coursing in a circular manner through urinary bladder into urethra**. Contrast, which was injected through the catheter, entered the urethra. The urinary bladder was not opacified by the contrast.

**Figure 3:**
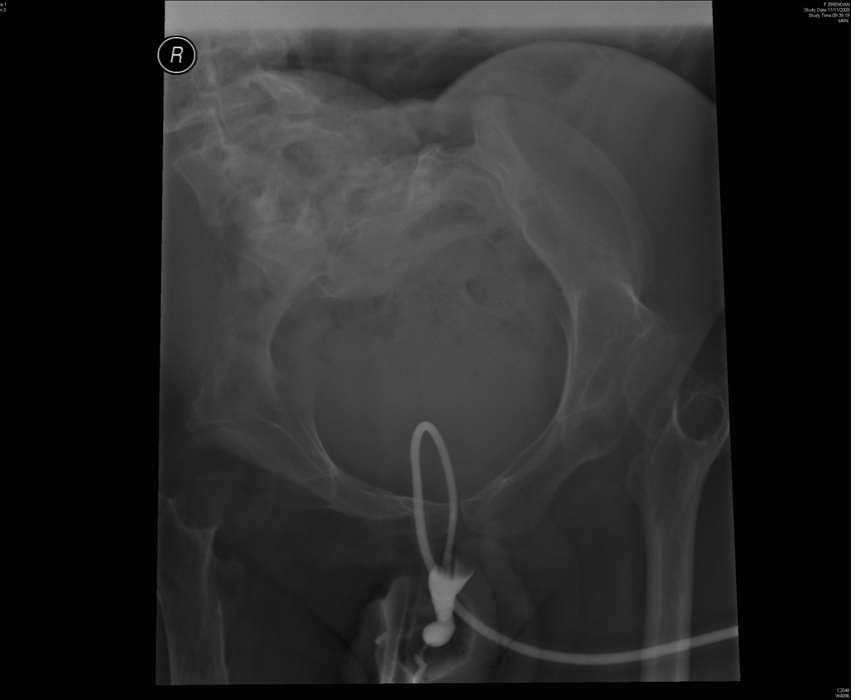
**Cystogram, performed on 11 November 2009, showed the contrast trickling from penis**. The tip of Foley catheter was located in urethra. The urinary bladder is not visualised by the contrast.

X-ray of pelvis was taken. This X-ray showed a soft tissue mass in pelvis, which was consistent with a distended bladder. (Figure 1) About twenty ml of contrast was injected through suprapubic catheter and X-rays were taken. The suprapubic catheter was patent; the catheter was not blocked. The Foley catheter could be seen going around in a circular manner through the urinary bladder into the urethra. The contrast did not opacify urinary bladder; but proximal urethra was seen. (Figure 2) The tip of Foley catheter was seen lying in proximal urethra. The balloon of Foley catheter had been inflated in urethra. Contrast trickled from penis. (Figure 3)

When the balloon of Foley catheter was deflated, this patient developed massive bleeding per urethra. A sterile 22 French Foley catheter was inserted through suprapubic track. The catheter drained urine, which was heavily stained with blood. He was admitted to spinal unit. This patient was administered intravenous infusion of 0.9% sodium chloride. He was prescribed meropenem one gram intravenously every eight hours for five days. He was advised to stay in bed until urine became clear. Haematuria subsided over a period of 48 hours. He did not develop high temperature or chills. The patient was discharged home a week later in a stable condition.

## Discussion

When a suprapubic catheter has been inserted inadvertently in to urethra going past the patulous bladder neck, the following signs will indicate incorrect positioning of Foley catheter.

(1) A very short length of Foley catheter will be outside the abdomen.

(2) If sterile saline is flushed through Foley catheter, sodium chloride solution may come out of external urethral meatus.

(3) When 30 ml of sterile 0.9% sodium chloride is injected with a catheter-tip syringe, there will be some resistance while injecting the saline through the catheter and it will not be possible to aspirate saline back into the syringe.

This case illustrates the importance of observing spinal cord injury patients for at least thirty minutes after changing a suprapubic catheter. Had this patient been kept under observation following change of suprapubic catheter, health professionals would have noticed that the new catheter did not drain any urine. Incorrect placement of suprapubic catheter could have been rectified without delay. This patient would not have developed retention of urine, severe discomfort in suprapubic region, and decompression haematuria.

Further, health professionals should be vigilant while inserting a catheter through suprapubic track. An experienced health professional would have noticed in this patient that following change of suprapubic catheter, only a very short length of Foley catheter was lying outside the bladder. Such an astute observation made by a senior health professional would have led to immediate repositioning of suprapubic catheter. This case reiterates the importance of delivery of health care by senior health professionals, who are conversant with the special situations that occur frequently in spinal cord injury patients, be it autonomic dysreflexia or pseudotumour in urinary bladder due to debris [2].

## Conclusion

This case shows that serious complications can occur during change of suprapubic catheter in patients with neuropathic bladder. After inserting a new catheter, health professionals should observe spinal cord injury patients for at least thirty minutes and ensure that (1) suprapubic catheter drains clear urine; (2) patients do not develop abdominal spasm or discomfort; (3) symptoms and signs of sepsis or autonomic dysreflexia are absent.

## Competing interests

The authors declare that they have no competing interests.

## Authors' contributions

SV developed the concept and wrote draft. PH reviewed medical images. All authors read and approved the manuscript.

## Consent

Written informed consent was obtained from the patient for publication of this case report and accompanying images. A copy of the written consent is available for review by the Editor-in-Chief of this journal.
